# Nocebo Effects on Muscular Performance – An Experimental Study About Clinical Situations

**DOI:** 10.3389/fphar.2019.00219

**Published:** 2019-03-11

**Authors:** Nina Zech, Milena Seemann, Magdalena Grzesiek, Anita Breu, Timo F. Seyfried, Ernil Hansen

**Affiliations:** ^1^Department of Anesthesiology, University Hospital Regensburg, Regensburg, Germany; ^2^Department of Anesthesiology, Klinikum Nürnberg, Nürnberg, Germany; ^3^Department of Anesthesiology, Sana Clinics Cham, Cham, Germany

**Keywords:** physician–patient communication, dynamometry, muscle strength, nocebo effects, informed consent, therapeutic communication, non-verbal suggestions

## Abstract

**Introduction:** Nocebo effects are not only seen in studies of pharmacology and placebo/nocebo research but also in clinical everyday situations. For generation of objective and quantitative data on the impact of negative communication we have evaluated the immediate effects of common sentences, non-verbal signals and situations in the medical context on muscular performance.

**Methods:** In an experimental study, 46 volunteers were tested by dynamometry of the deltoid muscle group to evaluate the maximal muscular strength during arm abduction. Baseline values were compared to performance after exposure to 18 verbal and non-verbal suggestions. Suggestions suspected to be negative were alternated with and compared to positively formulated alternatives.

**Results:** Verbal and non-verbal communication produced significant effects on muscular performance, resulting mainly in weakening. The decrease in muscle strength after risk information for informed consent (91.4% of baseline) was absent, when benefits of the treatment were named coincidently. The weakening effect of asking about “pain” and “nausea” (89.4%), and of the announcement of medical interventions (91.7%) could be avoided with alternative wording. Impairment of muscular performance was also observed with the nocebo-inducers negative memory (89.5%) or uncertain future (93.3%), in contrast to a positive memory or the orientation into the presence. Non-verbal suggestions like overhead anesthesia induction (89.9%), a transport in strict flat supine position (89.1%), or a view from the window to a parking lot (94.1%) significantly reduced maximal muscle strength, whereas face-to face induction, half-sitting position and a view into the landscape did not. 8 out of 9 tested clinical situations reduced maximal arm muscle strength significantly, whereas alternative formulations did not.

**Conclusion:** This study describes a quick, simple and uniform test using objective measurement of maximal muscle strength to allow for identification, quantification, and comparison of negative suggestions, regardless of their specific content and effect. Muscle strength is a clinically relevant parameter with regard to early mobilization, risk of falling and sufficient breathing. Furthermore, the observed impairment of muscular performance could reflect a general “weakening effect” of negative suggestions. In addition, the test facilitates development and verification of appropriate alternatives to prevent nocebo effects in patients, thereby improving patient communication.

## Introduction

Health care providers affect patients and their healing not only with medication, treatments and interventions but also with their words and their personal appearance. Bernard Lown has stated: “Words are the most powerful tool a doctor possesses, but words, like a two-edged sword, can maim as well as heal” (Lown, 1999). Words used in the communication with the patient not only have an impact on psychological phenomena such as pain, anxiety and stress, but also on autonomic body functions like circulation, peristalsis, wound healing, or immune reactions (Montgomery et al., 2002; Wobst, 2007). The medical environment is full of verbal and non-verbal signals that influence patients (in the following these are named “suggestions” as used in placebo research). Inadvertently, most of these suggestions are negative. By eliciting negative expectations resulting in nocebo effects, or by using words that directly affect patient perception, these suggestions can interfere with the treatment and the healing process (Lang et al., 2005; Häuser et al., 2012b; Benedetti, 2013; Hansen and Zech, 2019).

Communication in the medical context affects pain, stress and anxiety. In a study, announcing a painful intervention paired with empathetic statements did not decrease but rather increase pain and anxiety (Lang et al., 2005). The use of negative words was identified as the reason for this unexpected finding and disproof of a common expectation. A number of studies have shown that “painful” words can increase pain, supporting the conclusion that words can hurt (Ott et al., 2012). Pain was also significantly increased after explanation of the local anesthesia, prior to spinal or epidural puncture, by induction of nocebo effects and the use of negative words (Varelmann et al., 2010). Similarly, talking about nausea can induce nausea (Colagiuri and Zachariae, 2010). Other suggestions have been reported to impact body functions and processes and thus may interfere with treatment and therapeutic success (Barber, 1965; Häuser et al., 2012a; Benedetti, 2013; Zech et al., 2014). An especially relevant and important issue with respect to negative suggestions and nocebo effects is the medical informed consent (Miller and Colloca, 2011; Häuser et al., 2012b; Cohen, 2014; Colloca, 2015, 2017; Zech et al., 2015). Numerous articles describe the triggering of nocebo effects via generation of the expectation of a negative outcome (Colloca and Miller, 2011; Benedetti, 2013; Hansen et al., 2017). Presenting information in an insensitive way can induce exactly the specific side effect addressed (Häuser et al., 2012b).

Most patients experience medical settings as a serious and critical situation and, to some extent, as an existential threat. In such situations, individuals (not unlike animals) tend to enter a trance-like altered state of consciousness. Accordingly, surgical patients may behave as though hypnotized (Cheek, 1962). One of the essential characteristics of this natural trance state is a heightened focus of the patients with a strong tendency to refer all incoming information and signals to themselves. Another characteristic with high clinical significance is the increased suggestibility enhancing the impact of suggestions (Hansen and Bejenke, 2010).

Innumerable examples of specific effects resulting from specific suggestions have been documented, such as salivation when hearing the word “lemon” or local anesthesia with the suggestion of immersion in ice-cold water (Barber, 1965). Many effects of suggestions in the clinical setting, however, are difficult to trace or demonstrate in a timely manner. This complicates identification and avoidance of negative suggestions, and development of better alternatives. Similarly, although it is clear that with any medication or surgery the placebo effect should be utilized to increase therapeutic efficacy (Benedetti, 2013), optimization of such “open” pronouncements is only beginning and is complicated as long as any suggestion is tested by its specific effect.

Therefore, we examined a variety of stimuli from daily clinical practice prompted by words, whole phrases, imagination of situations, or non-verbal, visual stimuli in an experimental study for their immediate effects on one uniform parameter, namely maximal muscular strength. The latter represents a feasible valid parameter of physiology research with clinical relevance, yet rarely used in the context of communication. The primary aim of the study was to assess whether suggestions from the medical setting affect maximal muscular strength as measured by dynamometry, to detect possible nocebo effects. With this uniform tool a wide spectrum of relevant and common suggestions of the medical context were tested including the personal introduction of the physician, the assessment of symptoms, the risk information in order to gain informed consent, the view from the patient’s room, the transport in the hospital, announcing an intervention, the anticipation of the treatment, the induction of anesthesia, and the frequent negative memories of illness and treatment attempts. For these triggers assessed as negative alternatives were formulated and tested for comparison. Sensitization for nocebo effects in clinical everyday communication and demonstration of its avoidance by different formulations could show a possible way to improve healthcare provider-patient communication.

## Materials and Methods

### Design and Participants

After approval by the local ethics committee (EC University of Regensburg, 13-101-0030), an experimental study was performed with 46 volunteers after informed consent. The age of the participants was limited to 18–70 years, and their occupations were limited to non-medical professions. The reason for the accepted wide range of age was to facilitate translation of results to the clinical situation of patients in hospitals. Test persons were recruited through announcement to visitors of the hospital, friends and acquaintances. Participation was without financial compensation. Every participant was tested by the same tester (MG). Testing time was 80–90 min.

### Measurement of Maximal Muscle Strength Under Suggestions

**FIGURE 1:**
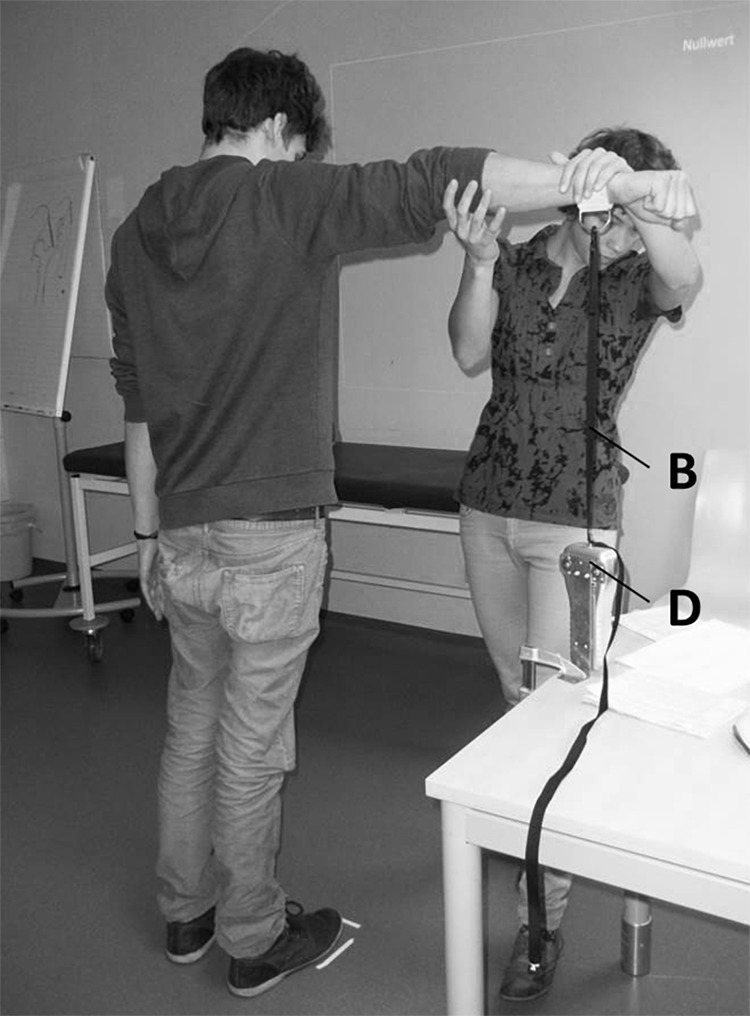
Tester positioning test person for dynamometry of arm abduction (the picture showing the tester, MG and a test person that both gave permission was taken by one of the authors, EH). D, dynamometer; B, band connecting dynamometer and wrist.

Isometric contraction of the deltoid muscle group during arm abduction was tested by dynamometry. In a neutral seminar room with a beamer the test person was placed in a defined upright position with the dominant arm stretched out laterally at an angle of 90°. A dynamometer (FORCE GAUGE FM200, PCE Deutschland GmbH, Meschede, Germany) was connected with a band to the wrist and the hand formed a fist for measurement of maximal muscular strength in arm abduction for 3 s (Figure 1). The dynamometer has a capacity of 196.0 N, a resolution of 0.05 N and was set to the peak hold mode. Due to the high variance of muscle strength between individuals, results were expressed in percentage of the baseline value that was determined in 10–12 measurements for each test person. With regard to the individual tested maximal muscle strength measured under these conditions is a rather robust physiological parameter, as confirmed in this study with a variation of ±6.3% for the baseline, comparable to reports on the hand grip test in clinical neurologic practice (Bohannon and Schaubert, 2005). The conversion of absolute values to relative values is a common method in physiology research and especially dynamometry to correct for differences in baseline, as known for muscle strength of study participants of different sex or age.

### Tested Suggestions

Suggestions were presented in form of words, sentences, situations and non-verbally as pictures or video clips. Three blocks of 10 words each were tested (Table 1). The wording of the sentences and the descriptions of the faced situations are given in Table 2. They aim to represent various clinical situations a patient might be confronted with during a hospital stay. Also the remembrance of a negative past (e.g., by anamnesis) and the anticipation of an uncertain future (e.g., surgery ahead) are typical clinical situations for patients. The non-verbal suggestions are illustrated in Figure 5. Those representing “transport to the OR” comprised video clips, version A the patient in strict supine position, version B with raised head section. Always pairs of a suggestion were tested: a version A (presumably negative) and a version B (presumably positive).

**Table 1:** Lists of words tested for effect on maximal arm muscle strength.

Words		Maximal strength (%)	Significance
Positive	Support, healing, health, confidence, care, help, mindfulness, wellbeing, company, strength	101.6 (92.6, 106.5)	ns
Negative	Pain, paralysis, blood, syringe, put to sleep, death, operation, accident, illness, nausea	102.0 (92.1, 110.1)	ns
Neutral	Nurse, monitor, doctor, ECG, stretcher, medicine, infusion, bed, white, name plate	100.9 (94.6, 106.9)	ns

*Median (in % of baseline) and interquartile range. Ns, not significant according to Friedman test.*

**Table 2:** Wording of the negative (Version A) and positive (Version B) suggestions, representing every day clinical situations.

**Giving cheer and support to a patient**	
Version A	You don’t need to be afraid. Don’t worry.
Version B	We are right by your side until you have successfully finished your procedure.
**Introduction of the physician (anesthetist)**	
Version A	Hallo, I’m Dr. Smith. I’ll put you to sleep now. We’ll start with the first drug, which will make you feel drowsy or drunk. Now we’ll start the second drug, which will burn a little bit. It will be all over soon.
Version B	Hallo, I’m Dr. Smith, your anesthetist. I’m here for your comfort and your safety. We are starting with a strong analgesic now that will make everything easier. Now I am giving you the second medication that will induce a restful sleep. I will be right by your side until you have finished your procedure successfully.
**Evaluation of symptoms in the recovery room**	
Version A	Let us know, when you feel pain. Do you feel nauseous?
Version B	Let us know, if there is anything to make you feel better. We always can do something good for you. Do you feel okay?
**Risk information informed consent**	
Version A	If you wish, we can place a pain catheter, with the risk of infection, allergic reaction, and damage to blood vessels or nerves.
Version B	We have the option of a catheter to prevent discomfort. Even though there is a risk of infection, allergic reaction, or damage to blood vessels or nerves you will have to take fewer pills, are more mobile, feel and recover better, and perhaps can go home sooner.
**Memories and expectations**	
Negative past	Remember a situation, where something went really wrong. Everybody was disappointed in you, including yourself. It was terrible. You were really ashamed.
Positive past	Remember a situation, when you were really successful and entirely satisfied with yourself. Everything went so well – totally perfect.
Negative future	Imagine an uncomfortable situation is about to take place: an impending operation, a performance review with your boss, an exam, or a confrontation with your partner. The result is uncertain.
Presence	You are fully in the here and now. You can feel the solid ground under your feet, notice your breath and your upright position while your mind is clear and open.

### Application of Suggestions

Participants listened to recorded instructions explaining the defined position and the sequential procedure of the muscle test, whereas suggestions were given verbally face-to-face. Visual suggestions included pictures or video clips projected on the wall in front of them. First, the baseline was established by 6 measurements after the verbal instruction. As it is shown in Table 3, each suggestion was given after an appropriate introduction. With the command “Now 1-2-3” explosive muscle contraction are avoided to allow for measurement of muscle strength in contrast to muscle power. Tests were separated by breaks, arithmetical tasks and repeated determinations of blank values. To prevent incorrect measurements because of exhaustion an additional break was inserted whenever a baseline value fell below 90% of the previous, and the test repeated subsequently. Preliminary tests had shown strong impact of test order, namely stronger weakening when a negative suggestion was followed by another negative one. Therefore, general randomization was waived and presumed negative (version A) and presumed positive suggestions (version B) were alternated to avoid accumulation effects. Randomization was limited to the order of the 9 themes. Since for any negative suggestion taken from everyday clinical practice an alternative version was generated, direct in-pair comparison was one aim of the study.

**Table 3:** Wording for introduction of the given suggestions.

Suggestion	Introduction
Baseline	“Now pull upward with maximal power. Now 1-2-3.”
Words or sentences	“Again, stand upright, lift your arm. Close your eyes and imagine you are a patient in a hospital. You are faced with the following words/sentences. Take your time and let it affect you, and then pull upwards as hard as you can...Now 1-2-3.”
Situations	“Again, stand upright, lift your arm. Close your eyes and imagine the situation I suggest to you. When you are there, please nod and then pull upwards as hard as you can…Now 1-2-3.”
Visual suggestions – Anesthesia induction (pictures) – Transport to OR (video clips) – View from patient’s room (pictures)	“Again, stand upright, lift your arm. Imagine you are patient in a hospital, and… – …you are in the OR and waiting to get your anesthesia, – …you are taken from the ward to the OR in your bed, – …you are looking out the window from your room. Let the impression affect you, and then pull upward as hard as you can…Now 1-2-3.”

### Statistical Analysis

With non-normal distribution of the relative values of muscle strength after suggestions results were reported as median and interquartile range. Groups of baseline, version A and version B were compared using the Friedman two-way analysis of variance by ranks. In case of significant findings the Wilcoxon rank-sum test with Bonferroni–Holm correction was applied. Statistical significance was assumed as *p* < 0.01.

## Results

### Baseline Muscle Strength

The mean age of the test participants was 34.3 ± 15.2 years, with a range of 19–70 years (medina 28 years). 25 women (54.3%) and 21 men (45.7%) took part, none of the participants had a medical background, like working in hospital, studying medicine etc. The first language of every participant was German. In the muscle strength test, reproducibility of the blank values (10–12 measurements) for a given individual was high, with a standard deviation of 6.3%. The absolute values varied considerably between participants, ranging between 26.5 and 135.2 N with a mean of 64.7 ± 25.5 N.

### Effects of Words and Sentences

**FIGURE 2:**
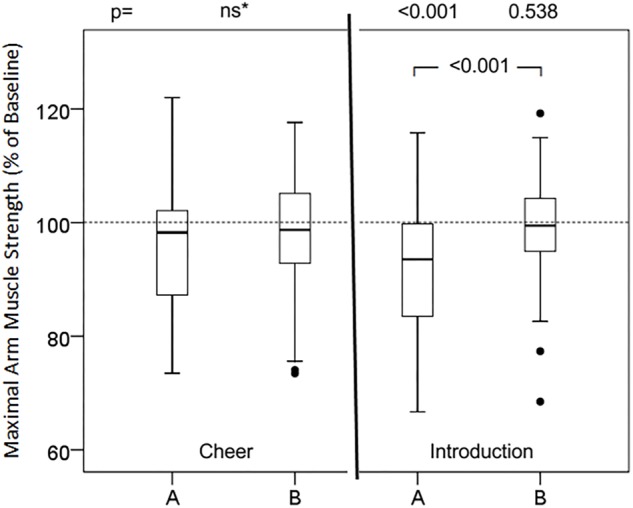
Effects of cheer and introduction in two versions on maximal arm muscle strength. After baseline dynamometry of arm abduction a sentence of cheer or introduction was presented and maximal muscle strength measured again. Box- plot diagrams of relative values are given, comparing version A and version B with the baseline value and versions A and B among each other. ^∗^Friedman test; ns, not significant; *p*, according to Wilcoxon test.

**FIGURE 3:**
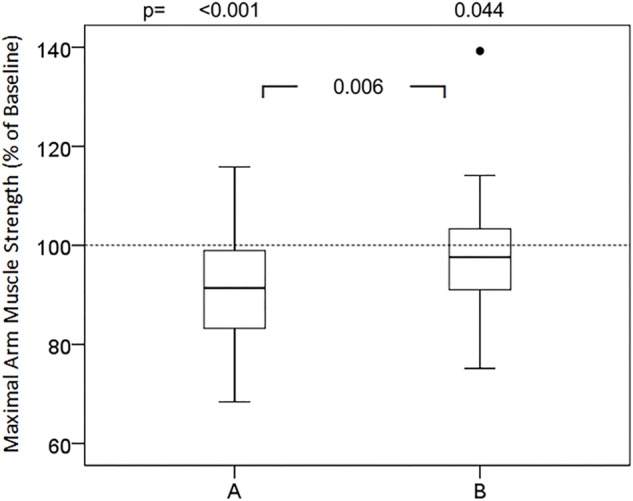
Effects of symptom evaluation in two versions on maximal arm muscle strength. After baseline dynamometry of arm abduction a sentence of symptom query was presented and maximal muscle strength measured again. Box- plot diagrams of relative values are given, comparing version A and version B with the baseline value and versions A and B among each other. *p* according to Wilcoxon test, when Friedman test significant.

Groups of presumably positive, negative and neutral words did not cause significant changes in relative maximum muscular strength (Table 1). In contrast, almost all presumably negative sentences (versions A) induced statistically significant attenuation of maximum muscle strength of the arm (Figure 1–3). However, none of the tested phrases that are commonly used to make the patient feel at ease (versions B) raised muscular performance above baseline values. In detail, the suggestions of cheer (Figure 1) did not affect muscle strength significantly, neither version A (98.2%; 87.2, 102.4, ns) nor version B (98.5%; 92.5, 105.7, ns). The words of the doctor to present himself to the patient and during induction of anesthesia (Figure 1) significantly reduced muscle strength in version A (93.5%; 83.4, 99.9, *p* < 0.001), while version B was neutral compared to baseline (99.4 ± 9.9%; 94.4, 104.4, *p* = 0.538) and significantly different to version A (*p* < 0.001). The evaluation of symptoms, namely pain and nausea (Figure 2), attenuated maximal arm muscle strength significantly in version A (91.4%; 83.0, 99.3, *p* < 0.001), but not in version B (97.6%; 90.7, 103.5, *p* = 0.044), with highly significant difference between the versions (*p* = 0.006). Giving risk information (Figure 3) caused marked weakening in version A (91.8%; 84.7, 98.7, *p* < 0.001) but no significant effect in version B (96.4%; 91.7, 105.9, *p* = 0.165), with a highly significant difference in the effect of the versions (*p* = 0.002).

### Effects of Situations

**FIGURE 4:**
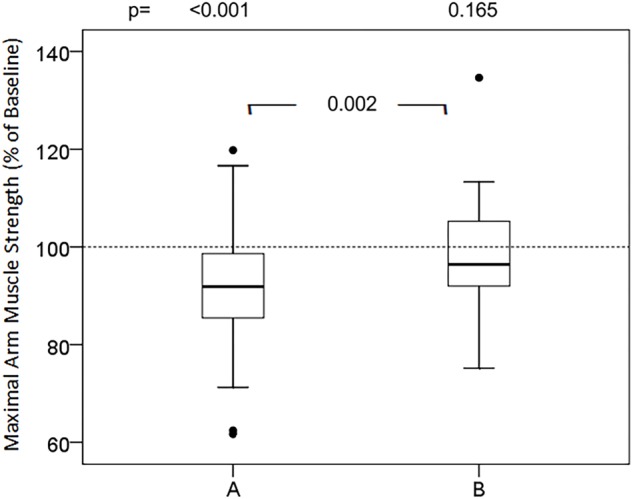
Effects of informed consent in two versions on maximal arm muscle strength. After baseline dynamometry of arm abduction a sentence of risk information was presented and maximal muscle strength measured again. Box- plot diagrams of relative values are given, comparing version A and version B with the baseline value and versions A and B among each other. *p* according to Wilcoxon test, when Friedman test significant.

The strongest responses were observed with suggestions of specific situations and conditions (Figure 4). Recall of a negative memory (89.4%; 79.9, 97.6, *p* < 0.001) and the suggestion of an impending negative situation (93.3%; 86.2, 98.0, *p* < 0.001) resulted in a statistically significant impairment of muscular performance. On the contrary, recall of a positive, encouraging memory (100.7%; 93.8, 110.0, *p* = 0.320) did not impair muscle strength, the difference in the effects of negative and positive memory being highly significant (*p* < 0.001). The orientation to the presence and bodily sensations led to some weakening (95.4%; 90.8, 103.3, *p* = 0.024), but with a strong trend to a lower effect than with expecting a negative future.

### Effects of Non-verbal Suggestions

Also non-verbal suggestions, namely the versions A of the presented images (Figure 6), caused decreases in muscular performance, whereas the alternative versions B produced no “positive” results but rather kept muscle strength unimpaired (Figure 7). In particular, the overhead look of the doctor during anesthesia induction (91.0%; 82.7, 94.7, *p* < 0.001) resulted in a significant loss of muscle strength, in contrast to the face-to-face position (96.8%; 90.4, 103.4, *p* = 0.057), representing a highly significant different reaction. The video clip of transport in strict flat supine position (89.3%; 83.2, 97.1, *p* < 0.001) significantly impaired muscle performance, whereas the half-sitting position (97.8%; 91.4, 102.6, *p* = 0.071) had no weakening effect. The view from the patient’s room to a parking lot (94.1%; 80.9, 99.9, *p* < 0.001) reduced maximal muscle strength significantly, in contrast to the view into the landscape (96.6%; 91.3, 102.0, *p* = 0.045).

**FIGURE 5:**
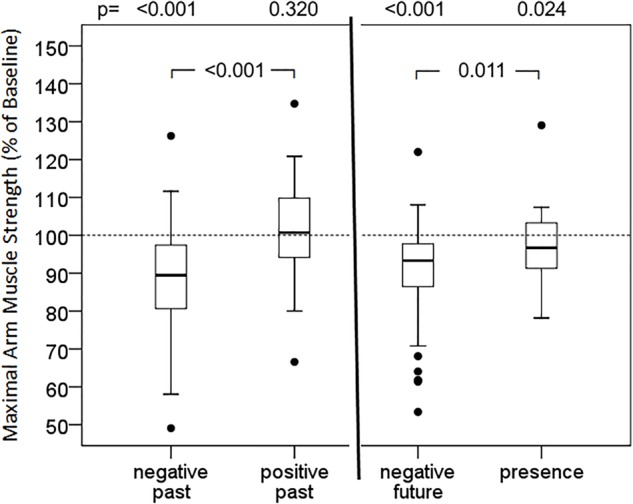
Effect of memories and expectations on maximal arm muscle strength. After baseline dynamometry of arm abduction test persons (*n* = 46) focused on negative or positive past, uncertain future or presence, and maximal muscle strength was measured again. Box- plot diagrams of relative values are given, comparing every situation with baseline values and the situations among each other. *p* according to Wilcoxon test, when Friedman test significant.

**FIGURE 6:**
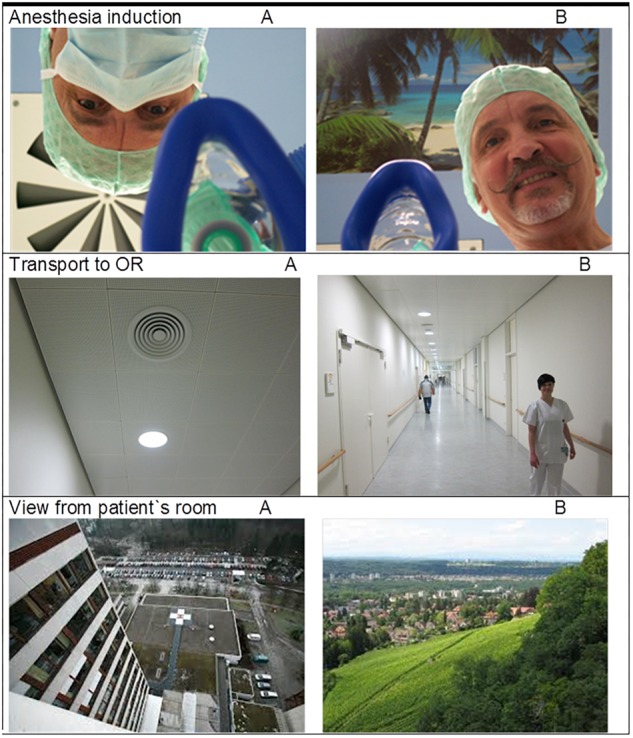
Non-verbal signals in two versions (all 6 pictures were taken by one of the authors, EH; the upper two pictures show one of the authors, EH; the persons visible in picture “transport B” gave permission).

**FIGURE 7:**
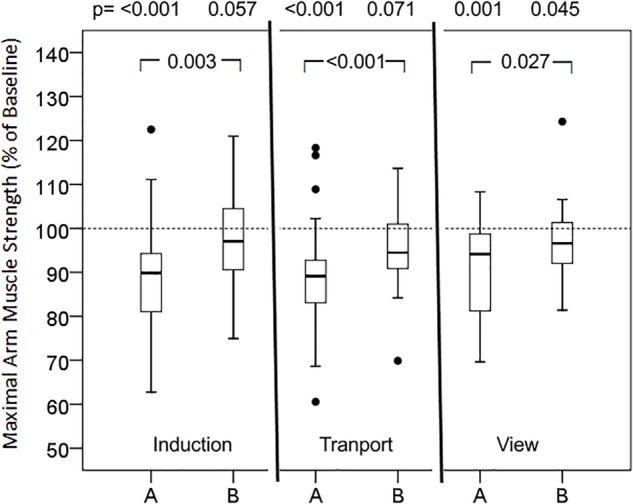
Effects of non-verbal signals in two versions on maximal arm muscle strength. After baseline arm dynamometry test persons (*n* = 46) saw a picture or a video clip (Transport), and maximal muscle strength was measured again. Box- plot diagrams of relative values are given, comparing every picture with the baseline value and versions A and B among each other. *p* according to Wilcoxon test, when Friedman test significant.

### Influencing Factors

The weakening effects of suggestions in version A were not normally distributed indicating that some test persons showed an especially strong reaction. Therefore, all interventions that have resulted in a significant impairment of the muscular performance were analyzed for influencing factors. In cubic regression analysis a slight dependency of age (*R*^2^= 0.26, *p* = 0.016) was found with a lower response in the young adults. There was no difference in the reactions of men (92.6%) and women (92.0%). With a Spearman Rho of *r* = -0.075 the correlation to the individual suggestibility score Harvard-Group-Scale-of-Hypnotic Suggestibility (Shor, 1962; Miller, 1980) (data not shown) of the test persons was minimal and statistically not significant (*p* = 0.065). In some of the tested participants individual experiences with illness and treatment could be tracked as a possible explanation of an unexpected reaction, such as an activation following negative words.

## Discussion

### Originality and Impact of the Study

The results of this study demonstrate significant immediate effects on muscular strength of both verbal and non-verbal suggestions that did not have a direct relation to the context of muscle work, muscular performance or movement. From sports medicine it is well known that cognitive strategies like imagery of performance, self-talk, preparatory arousal, and goal setting (Tod et al., 2015), or hypnosis (Barber, 1966), or positions like power-posing (Carney et al., 2010) can increase strength performance. In addition, several studies have tested and demonstrated modulation of the motor system by placebo and nocebo effects (Kalasountas et al., 2007; Bottoms et al., 2014; Carlino et al., 2014; Stoekenbroek and Kastelein, 2017; Butera et al., 2018; Fiorio, 2018). Again, those studies intended to affect sport performance, muscular training, or side effects and diseases that impair movement and muscular functions. Accordingly, the suggestions inducing expectations were directed toward muscular strengthening or against muscular impairments, respectively. The present study did not contain suggestions related to movement or muscular performance. Instead, the observed effects on maximal muscle strength originated from words and signals taken from the medical setting and daily clinical practice.

In most former studies subjective measures like pain or comfort scales were used to quantify effects of health care providers and medical environment on patients as negative or positive (Barber, 1965; Varelmann et al., 2010). In the present study objective data are obtained by using dynamometry, an established measurement system of physiology. This also allowed quantification of suggestion and nocebo effects.

Suggestions, on the other hand, generally are evaluated for their specific effects (Barber, 1965), e.g., phrases containing the word “pain” for induction or aggravation of pain (Benedetti et al., 2007; Varelmann et al., 2010). In contrast, this study used one general parameter, namely maximal arm muscle strength, to test suggestions of different content and context, thus facilitating comparison of the induced nocebo and placebo effects. To our knowledge this is a novel approach to test for placebo/nocebo effects. Usually specific effects, e.g., an increase in pain or in nausea, are monitored and can only be compared by their effect sizes. The use of one unique parameter for different suggestions described here allows for direct comparisons.

This study describes a research tool to test changes in maximal muscle strength in response to various suggestions. The chosen parameter is the result of an immediate reaction. By contrast, many nocebo effects to risk information for instance take time to precipitate and to be evaluated. The changes in maximal muscle strength as a timely response to suggestions together with the good comparability by one common target parameter make development and testing of modification possible. By introducing the option of timely comparisons the technique represents a novel approach to investigate and develop more helpful alternatives to inadvertent negative suggestions that can adversely affect patient care, thus improving doctor–patient communication.

### Suggestions of Medical Communication Can Weaken Patients

Lists of words showed no significant impact on muscular performance. Only in the context of sentences or instructions do they seem to gain relevant meaning and significance, and can become inducers of expectations and eventually of placebo or nocebo effects. Also the suggestions of cheer (see Figure 1, left side) had no significant effect. It has been proposed that with formulations containing strong words, like here “afraid” and “worry” in version A, together with a negation the former cannot be neutralized. The reason is the limited efficacy of negations versus strong suggestions (Tettamanti et al., 2008; Armstrong and Dienes, 2013). In this study, neither an enhancing effect of such a common cheer, nor a weakening effect of the included negative words could be demonstrated. The well-intentioned advice “Don’t worry!” could be considered a trigger for a positive expectation and thus an inducer of a placebo effect. At least in the muscle test these encouraging words had no positive effect.

During personal introduction and announcement of the interventions for induction of anesthesia words with negative connotation obviously were responsible for the observed decrease in maximal muscle strength with version A. On the other hand, inclusion of positive words like “comfort,” “safety” and “be by your side,” most important for anxious patients to hear, are able to neutralize this weakening effect (see Figure 1, right side). Explaining the side effects of medication used for induction of anesthesia such as fentanyl and propofol is necessary and common practice but can induce the corresponding expectancy and subsequently a nocebo effect. The information can be expressed less specifically in order not to induce exactly the sensations of dizziness and nausea, or burning pain (propofol phlebalgia) addressed (Hansen and Bejenke, 2010).

The theme of the suggestions in Figure 2 is the common and in medicine frequently necessary question about symptoms, where often negative terms are applied. Of these, “pain” is one of the most often used words with negative connotation in medical treatment. Within a few minutes a patient in the recovery room can be exposed numerous times to this word. The magnitudes of placebo and nocebo effects in pain are high and comparable (Petersen et al., 2014). Their mechanisms have been evaluated and elucidated to quite some extend (Amanzio et al., 2001; Benedetti et al., 2007). A considerable number of studies have reported that words like “pain” can increase pain (Lang et al., 2005; Varelmann et al., 2010; Ott et al., 2012). In addition, they can weaken the patient as demonstrated in this study. Obviously, this effect can be prevented by using alternative expressions with positive connotations as version B in Figure 2. This highlights the importance of a careful choice of words in patient communication. Asking about pain and pain scores adversely affects patient’s reports and post-operative experiences, but can be used interchangeably with inverted comfort scores (Chooi et al., 2013). Sometimes it is argued that we really need information about pain and that information might be lost with the alternate question about comfort. But a patient in pain will name this pain. However, for the induction of a suggestion or nocebo effect it makes a big difference whether the word pain comes from the patient himself or from outside (Hansen and Zech, 2019, this issue).

Risk information for informed consent can induce the symptoms addressed (Wells and Kaptchuk, 2012; Häuser et al., 2012b; Colloca, 2015). In this study, not the specific side effects but a significant impact on muscular performance is documented (Figure 3). It is notable that the same risk information, when shared in connection with positive aspects (version B), lacks this depressive effect. The results provide evidence that the wording is essential, and no omission or whitewash of risk information is necessary. The neutralization was produced by the addition of positive aspects, here the intention of the therapy and the benefits expected. Other options include the positive connections to the measures taken for prophylaxis of side effects, the monitoring and early detection to initiate immediate treatment options, and the possible patient’s own contributions to prevention (Hansen and Zech, 2019, this issue). Perhaps these positive suggestions generate positive expectations and thereby compete with the negative expectations and nocebo effects induced by the risk information. In addition, only the simultaneous presentation of risk and benefit enables the patient for a balanced consideration and a sound decision, the basis of informed consent (Zech et al., 2015).

The strongest reactions were observed with the suggestion of a specific situation or condition. Negative memories (Figure 4, left side), reactivated, stressed and consolidated during diagnosis, interviews and evaluation of the medical history, are typical situations for the patient. The problem-focused, deficit-based mode of operation in medicine forces and holds the patient in this negative, fear-based state and therefore weakens the mental and physical condition of the patient. The speed of onset and the extent is remarkable. A voluntary movement was diminished within less than a minute, and by up to 50%. The recollection of diseases, symptoms and treatment attempts is a common origin of nocebo effects (Colloca and Miller, 2011; Häuser et al., 2012b). Remembering a positive past did not trigger an increase in muscular performance in this study, but at least was able to neutralize the effects of a negative memory. As an impending event is approaching, such as an operation or a treatment with an unclear outcome, the perception in itself weakens the patient, as was demonstrated in this study (Figure 4, right side). This situation describes a clinically quite common and relevant situation, again prone to trigger nocebo effects. An option to leave the burden of bad experiences or the expectation of an unfavorable future is to focus on the present, the here and now, as taught in mindfulness-based therapies (Sipe and Eisendrath, 2012). The concentration on both, the physical presence and the present moment to some extend reversed the impairment induced by the suggested condition of uncertainty (Figure 4, right side).

Evaluating non-verbal suggestions, the standard situation of the induction of anesthesia, showed negative effect (see Figure 6). The image of the doctor’s face upside-down, hidden behind a mask, interferes with biologically based face perception and recognition (McKone et al., 2012), and might even induce dizziness and nausea. In the fearful moment of losing consciousness the patient is not faced with a human face and being. Actually, there is no medical need for this position at this moment, and holding the mask for pre-oxygenation can easily be performed face-to-face. Hygienic concerns about temporarily lifting the mask can be dispelled, and the ceiling (the dominant perspective of the patient) can be decorated with posters that inspire dissociation to a “safe place” (image “anesthesia induction B” in Figure 5). This change in perspective and reduction of negative signals immediately restored muscular performance. Similarly, transportation of the patient in a bed is dominated by the view to the ceiling and concerns thousands of patients every day. Rarely is there an indication for a strictly flat supine position. The patient is not able to change this dreadful condition, and requires a careful, compassionate nurse to change it for him or her. Finally, the perspective from the patient’s room represents a long-term factor of influence. Faster recovery and fewer analgesic doses have been reported for patients on the same ward after cholecystectomy depending on whether the windows were facing a brick building wall or a small stand of deciduous trees (Ulrich, 1984).

### Limitations of the Study

The limited number of test persons might have prevented significant positive effects of certain “positive” suggestions. On the other hand, negative words and phrases might elicit stronger effects in patients than in healthy volunteers studied here. However, a pilot study seemed appropriate before testing patients and before dealing with the constraints of hospital settings. A subsequent study on patients is in progress. Besides the clinical situation of patients anxiously awaiting an operation, it would be interesting to evaluate the effects in patients under limited physical condition. A weakness of the present survey is the limited randomization of the order of tested suggestions, where the themes were randomized, but not the sequence of version A followed by version B. This was owed to the previous observation that accumulation of positive or negative suggestions distort the individual effect, and to the objective to directly compare two versions of the same topic.

The physiology behind the observed changes in muscular performance is unclear and probably not uniform. The impact of psychological processes on muscle function and motion is manifold. Language-induced motor activity, arousal and affirmation effects, modulation of motor cortex or cortico-spinal excitability, and many more may play a role (Li et al., 2004; Pulvermuller et al., 2005). In this study, changes in muscular performance were not the results of priming, learning, conditioning, mental training or psychotherapeutic interventions, but immediate and direct reactions directed by words or images, by expectations or by the condition itself (Fiorio et al., 2014). The physiological mechanisms of the observed effect is unknown. Many mechanisms are proposed and discussed for the psychological and physiological responses of communication according to the many fields of research like ethology, behavioral and communication research, psychosomatics, hypnosis and placebo research. Even the latter describes various factors possibly involved like hormones, immune mediators, endogenous opioids, dopamine and other neurotransmitters, and local changes in brain metabolism, microcirculation and neural functions (Benedetti et al., 2003; Finniss et al., 2010; Benedetti and Amanzio, 2013). So “there is not one placebo effect, but many.” Different mechanisms have been described for expectation- or conditioning-induced placebo effects (Amanzio and Benedetti, 1999). In addition, different mechanisms, different neurotransmitter involvement (Scott et al., 2008), and the activation of different brain areas (Freeman et al., 2015) have been demonstrated for placebo and nocebo effects, the latter with greater significance for this study here. Similarly, various and different mechanisms are discussed for the effects of suggestions in hypnosis (Barber, 1965; Faymonville et al., 2000; Jensen et al., 2015; De Benedittis, 2015). But most relevant to the results described here is research specifically on motor functions (see above). In preliminary experiments on increased corticospinal excitability examined with transcranial magnetic stimulation (data not shown) we found no evidence of an impact of the suggestions evaluated here on nerve conduction. More plausible are central nervous effects.

### Clinical Implications

The clinical relevance of this study is founded by the tested wide spectrum of suggestions from daily clinical practice. In addition, impairment of muscular performance has far-reaching clinical impact on patients. It could foster the development of pneumonia, cause fracture after stumbles and falls, and impede or delay mobilization. Interestingly, the results may be utilized to improve mobilization, for instance by instructing a patient after hip surgery to focus on his former success in sporting events, or on enjoyable activities after rehabilitation, or on the here-and-now-present instead of the past, troublesome medical history. Basically, in this study the effect of each suggestion was measured against neutral baseline values. However, since negative suggestions alternated with positive suggestions, the results also provide evidence and assistance as to how to neutralize or even reverse prior negative effects. A remarkable result of this study is that, except in individual participants, no improvement of muscular performance was observed. An explanation may yield that the impressions in a hospital or medical practice setting are typically not perceived to be positive. Another reason might be that starting from an optimal condition as in healthy volunteers it is easier to impair maximal muscle strength than to foster it.

A further advantage of the described technique to measure and compare nocebo effects is that this way compound effects can be studied. Patients in clinical reality are not exposed to one single thread like pain or one single negative suggestion, but to a variety at the same time, for instance verbal and non-verbal stimuli, anxiety and pain. The compound action of many such suggestions then result in a compound effect that has much more to do with significant issues like immune responsiveness, healing or resilience that are hard to measure. The authors hypothesize that the demonstrated muscular weakening can stand for a more general “weakening effect” of nocebo suggestions and a common, clinically relevant “weakening” of patients in the medical setting. Further studies should test the correlation of the observed effects on maximal muscle strength with other parameters of patient competence and performance.

## Conclusion

The described method provides a unitary tool to test the effects of various suggestions. Current communication with patients relies on presumptions about the negative or positive nature of words, appearance and behavior of personnel, and suggestions originating from the medical environment. Objectification and quantification of the effects allows for comparison. The described testing system can be used as a tool to detect and study negative suggestions and nocebo effects, and to find better alternatives. Thereby the study may contribute to bridge neuroscience and everyday challenges of medical communication to improve clinical practice of healthcare provider-patient communication. Moreover, the used parameter maximal arm muscle strength during abduction might be a common surrogate marker for weakening effects on patients.

## Data Availability

All datasets generated for this study are included in the manuscript and/or the supplementary files.

## Author Contributions

EH contributed the study plan and design, supervision, literature search, data analysis, preparation of figures, tables and manuscript, and correction of manuscript. NZ and MS contributed the study design, application for ethic committee approval, literature search, participant recruitment, data collection and analysis, and preparation of the manuscript. MG involved the participant recruitment, data collection, and analysis. AB contributed the data and statistical analysis and preparation of the manuscript. TS contributed the literature search and correction of the manuscript.

## Conflict of Interest Statement

The authors declare that the research was conducted in the absence of any commercial or financial relationships that could be construed as a potential conflict of interest.
